# Chromatin Remodeling in Development and Disease: Focus on CHD7

**DOI:** 10.1371/journal.pgen.1001010

**Published:** 2010-07-15

**Authors:** Donna M. Martin

**Affiliations:** Departments of Pediatrics and Human Genetics, The University of Michigan, Ann Arbor, Michigan, United States of America; Medical Research Council Human Genetics Unit, United Kingdom

A central goal of developmental biology is to understand the mechanisms whereby undifferentiated, pluripotent cells differentiate into mature, organized cells and tissues. Recent advances in mouse genetics, embryonic stem cell manipulation, RNA inhibition, and genomics have now made it possible to explore these mechanisms with an unprecedented level of resolution. One major class of proteins, the chromodomain helicase DNA-binding (CHD) family of ATP-dependent chromatin remodelers, has emerged as important regulators of cellular differentiation. CHD proteins are thought to function in the nucleus via binding to DNA and regulating gene transcription. CHD7, a member of the CHD family, encodes a protein mutated in human CHARGE syndrome, a multiple anomaly disorder that affects hearing, vision, and cardiac, craniofacial, and nervous system development [1], [2]. Previous reports have shown tissue and developmental stage specific expression of CHD7 [3], [4], and there is evidence that CHD7 binds thousands of sites in the genome [5]. It is thus a major challenge to identify specific mechanisms of CHD7 action in pluripotency versus differentiation.

A study in this issue of *PLoS Genetics* [6] used genomics approaches to explore CHD7 binding sites and interacting partners in embryonic stem (ES) cells. The authors applied chromatin immunoprecipitation followed by next generation sequencing (ChIP-Seq) in ES cells under several different growth conditions. They found 10,483 chromatin sites bound by CHD7, most of which appear to be enhancer regions. CHD7 co-localized at these sites with potentially unique combinations of other DNA binding proteins, including p300, Oct4, Sox2, Nanog, Smad1, and STAT3 (see Table 1 for a list of genes and their official symbols; for the purposes of this Review, genes are referred to as cited in the original papers). In addition, genes directly regulated by CHD7 were subtly downregulated in ES cells, and these genes were far more ES cell–specific than either genes that did not change or those that decreased upon loss of CHD7. Despite the predominance of CHD7 binding sites in ES cell–specific genes, ES cell pluripotency, self-renewal, and reprogramming did not appear sensitive to CHD7 dosage. Thus, CHD7 seems to act as a rheostat of ES cell–specific gene expression without overtly controlling ES cell function.

**Table 1:** List of genes cited in the text and their official symbols.

Official Symbol	Protein Type	Aliases (mouse)
Ep300	E1A binding protein p300	KAT3B, **p300**
Pou5f1	POU class 5 homeobox 1	Oct-3, **Oct-4**, Otf-3, Otf-4
Sox2	SRY-box containing gene 2	Sox-2, lcc, ysb
Nanog	Nanog homeobox	ENK, ecat4
Smad1	MAD homolog 1 (Drosophila)	Madh1, Madr1, Mlp1, MusMLP
Stat3	Signal transducer and activator of transcription 3	Aprf
Smarca4	SWI/SNF related, matrix associated, actin dependent regulator of chromatin, subfamily a, member 4	**Brg1**, HP1-BP72, SNF2beta, SW1/SNF
Smarcc2	SWI/SNF related, matrix associated, actin dependent regulator of chromatin, subfamily c, member 2	BRG1-associated factor 170; **BAF170**
Smarcc1	SWI/SNF related, matrix associated, actin dependent regulator of chromatin, subfamily c, member 1	BRG1-associated factor 155; **BAF155**, Rsc8, SRG3
Smarce1	SWI/SNF related, matrix associated, actin dependent regulator of chromatin, subfamily e, member 1	**BAF57**; BRG1-associated factor 57
Pbrm1	Polybromo 1	**Pb1**; BAF180; Pbrm1
Arid2	AT rich interactive domain 2 (ARID, RFX-like)	zipzap/p200
Brd7	Bromodomain containing 7	BP75, CELTIX1, Ptpn13ip
Setdb1	SET domain, bifurcated 1	ESET, KMT1E
NLK	Nemo like kinase	AI194375
Pparg	Peroxisome proliferator activated receptor gamma	Nr1c3
Wnt5a	Wingless-related MMTV integration site 5A	Wnt-5a
Runx2	Runt related transcription factor 2	Cbfa1

*Genes in bold are aliases used in the cited references. Gene symbols are in reference to the mouse genome.

Like ES cells, neural crest–derived cells are pluripotent, since they differentiate into numerous cell types including craniofacial mesenchyme, heart, peripheral nervous system, and melanocytes. It has long been proposed that neural crest abnormalities contribute to the pathophysiology of CHARGE syndrome, but this had not been directly tested. In a related study, Bajpai et al. [7] explored CHD7 function in *Xenopus*, and found that CHD7 is vital for proper migration of neural crest in developing branchial arches. They also showed that human ES cells form a pluripotent population of cells that express markers of neural crest and can migrate to craniofacial mesenchyme and heart after transplantation into the chick neural tube. Migration of these neural crest–like cells (NCLCs) in vitro was also sensitive to CHD7 dosage. The authors concluded that CHD7 likely regulates cell migration in the developing neural crest.

**Figure 1:**
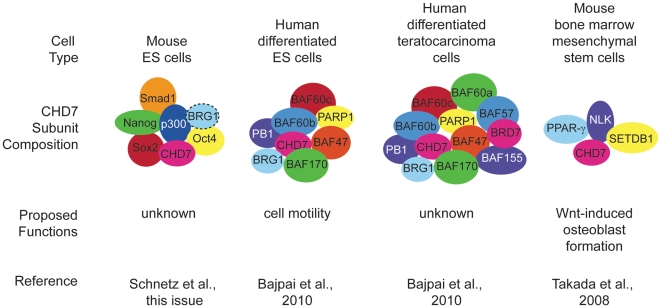
Cell type–specific DNA binding complexes containing CHD7. Proposed functions for CHD7 include cell motility and fate decisions. These functions appear to be unique to specific stem cell populations. Hatched lines around BRG1 in ES cells indicate that only some of the complexes have this protein as a component.

Because CHD7 binds to thousands of enhancer sites in specific tissues during development, it is possible that each site binds a unique protein complex whose composition changes over developmental time. Identification and characterization of these complexes is thus akin to separating the wheat (binding sites and complexes that have functional significance) from the chaff (binding sites and complexes that have no functional significance). In the paper by Schnetz et al., the “wheat” appears to be a set of about six factors (Smad1, Nanog, Oct4, Sox2, Stat3, and p300) that co-localize with CHD7 (based on ChIP-Seq data) at specific DNA binding sites called multiple transcription factor loci or “MTLs” [6] (Figure 1). Schnetz et al. also identified overlap between sites occupied by BRG1 and CHD7 in ES cells; however, the binding was not as extensive as for the other six factors [6]. Earlier data showed that BRG1 associates with OCT4, SOX2, and NANOG in ES cells [8], providing further support for the idea that CHD7 binding in ES cells may be generally associated with positive and negative regulation. In the study by Bajpai et al., the “wheat” comprises the BAF and PBAF chromatin remodeling complexes that belong to the SWI/SNF family [7]. They found, by co-immunoprecipitation in human NCLCs, that CHD7 binds numerous PBAF specific subunits, including BRG1, BAF170, BAF155, BAF57, PB1, ARID2, and BRD7 (see Table 1 for official symbols). Interestingly, a previous report showed that CHD7 forms a complex with SETDB1, NLK, and PPAR-gamma that binds to DNA in a Wnt-5a responsive manner and promotes Runx2 transcription during osteoblast formation [9].

**Figure 2:**
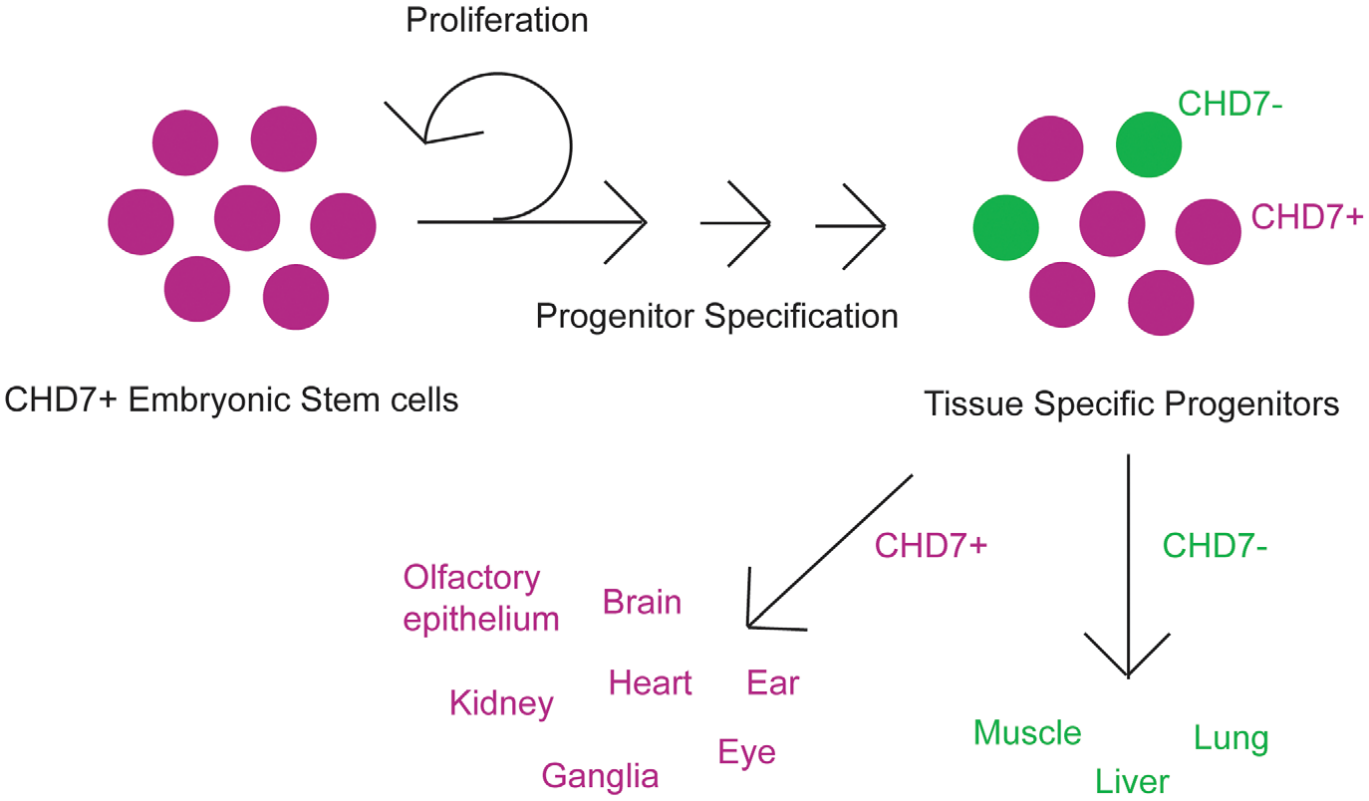
Cartoon schematic of CHD7 roles in cellular proliferation and/or differentiation. CHD7 is highly expressed in ES cells, and becomes restricted during development to tissue specific progenitor populations. These progenitors give rise to specific cell types in the various organs which are affected in CHARGE syndrome, including neural crest derivatives, central and peripheral nervous systems (ganglia), sensory organs (ear and eye), heart, and kidney. Some organs such as lung, liver, and skeletal muscle express no or very low levels of CHD7.

It is interesting to compare these recent and earlier studies for evidence of CHD7 function in pluripotency versus cellular fate determination and differentiation. CHD7 does not appear to regulate pluripotency in mouse ES cells [6] or human NCLCs [7], but is required for normal proliferation of olfactory neural stem cells [10]. CHD7 also seems to promote one or more aspects of cellular differentiation. Binding of CHD7-containing complexes activates osteoblast fates over chondrocyte or myocyte differentiation of bone marrow mesenchymal stem cells [9] and promotes migration of neural crest cells in the branchial arches [7]. These observations suggest multiple roles for CHD7 in regulation of cellular progenitor proliferation and/or differentiation (Figure 2). Further investigation is necessary to clarify whether CHD7 has similar or different roles in the various cell types in which it is expressed.

CHD7 and related proteins likely have unique functions in human development. Along with *CHD7* and the other eight *CHD* family members, there are about 30 mammalian genes encoding ATP-dependent chromatin remodeling proteins [11]. ATP-dependent chromatin remodelers differ from other chromatin modifiers (which control DNA methylation and histone acetylation) in that they are genetically non-redundant, haploinsufficient, and increase nucleosome mobility [11]. There are many human developmental disorders known to result from mutations in chromatin modifying proteins, including Rubinstein-Taybi (*CBP* and *EP300*), X-linked Alpha Thalassemia (*ATRX*), and Rett Syndrome (*MECP2*), among others [12]. Besides *CHD7* and CHARGE syndrome, there are few developmental disorders known to result from mutations in ATP dependent remodeling genes. Loss of *CHD5* may contribute to the microcephaly and physical features observed in Deletion 1p36 Syndrome [13], although its role is not yet established. Deletion of *WSTF* (a member of the ISWI class of ATP-dependent chromatin remodelers), along with several other genes in the critical interval on chromosome 7q11, results in Williams-Beuren syndrome, which presents with cardiac defects, craniofacial dysmorphisms, and developmental/cognitive impairments [14].

Accurate identification of CHD7 gene targets, interacting partners, binding sites, and complex subunit composition is going to require cooperative, detailed studies using high-throughput approaches and model organisms. One could argue that each cell type in a given tissue might have unique CHD7 binding sites and protein complexes that change over time. Finding these is a daunting task, and it seems more likely that common factors, targets, and molecular genetic pathways will emerge. It will be of great interest to know, for example, whether CHD7 regulates specific signaling pathways during organogenesis and whether those pathways can be manipulated by molecular intervention. In addition, there is no information yet on CHD7 protein domain-specific functions, over-expression phenotypes, or potential roles for CHD7 in cancer or aging. These topics are sure to occupy researchers for many years to come, and such efforts will likely allow us to harvest some wheat and make a nice, tasty loaf of bread.
